# Gene Influence in the Effectiveness of Plant Sterols Treatment in Children: Pilot Interventional Study

**DOI:** 10.3390/nu11102538

**Published:** 2019-10-21

**Authors:** Ismael San Mauro Martín, Elena Garicano Vilar, Sara Sanz Rojo, Luis Collado Yurrita, Eva Pérez Arruche, Esperanza Arce Delgado, Javier Andrés Blumenfeld Olivares

**Affiliations:** 1 Research Centers in Nutrition and Health, 28036 Madrid, Spain; info@grupocinusa.es (I.S.M.M.); elena@grupocinusa.es (E.G.V.); sara.sanz@grupocinusa.es (S.S.R.); 2 Medicine Department, Universidad Complutense de Madrid, 28040 Madrid, Spain; lcollado@med.ucm.es; 3 Hospital El Escorial, San Lorenzo de El Escorial, 28200 Madrid, Spain; research@grupocinusa.es (E.P.A.); nachespe@hotmail.es (E.A.D.); 4 Faculty of Medicine, Universidad Francisco de Vitoria, Pozuelo de Alarcón, 28223 Madrid, Spain

**Keywords:** children, cholesterol, genetics, low-density lipoprotein cholesterol, sterol

## Abstract

Cardiovascular disease is linked to high serum low density lipoprotein (LDL)-cholesterol levels. Cardiovascular risk may be indirectly influenced by genetic load. Serum LDL-cholesterol levels may be reduced by the consumption of food enriched with plant sterols (PS). The aim was to test a plant sterol treatment on cholesterol levels according to different genetic polymorphisms. A pilot interventional trial was performed in 26 children (*n* = 16 girls, *n* = 10 boys). Seven hundred milliliters/day of commercial skimmed milk with added plant sterols delivering 2.2 g plant sterols were ingested for three weeks. Blood draws were performed at the baseline and end of the study. Significant modifications of non-high density lipoprotein (HDL)-cholesterol (*p* = 0.010; *p* = 0.013) and LDL-cholesterol (*p* = 0.004; *p* = 0.013) levels appeared in the genes *LIPC C-514T* and *PPAR-α L162V* carriers. No statistically significant differences were observed for other genes. *LIPC C-514T* and *PPAR-alpha L162V* carriers could benefit from a plant sterol supplement to ameliorate hypercholesterolemia.

## 1. Introduction

Cardiovascular diseases (CVD) have gained relevance through the past years due to their importance in public health. They are the main cause of death around the globe, according to the World Health Organization [1].

Cholesterol levels are known determinants of cardiovascular health [2]. Atherosclerotic lesions start appearing in childhood, making cholesterol levels especially important on pediatric age [3].

Familiar hypercholesterolemia is the most prevalent genetic disorder among children and teenagers [4]. These children are a risk group for the development of CVD. Familial hypercholesterolemia results in alterations in carotid intima-media thickness, vessel walls and flow-mediated dilatation [2]. When strategies for lowering low-density lipoprotein cholesterol (LDL-cholesterol) levels are applied to these children, there is a deceleration in the progress of the lesions [5].

Since the modification of lifestyle factors is generally useful to control the lipidic profile, public health strategies base their recommendations on changes in dietary habits and physical activity [6]. Those dietary changes include limiting saturated fat and cholesterol intake [2]. Also, plant sterols (PS) seem to be beneficial for regulating hypercholesterolemia [7].

When a person ingests a daily dose of 1.5–2.4 g of PS, blood cholesterol may be lowered by 7–10.5% within 2–3 weeks, according to EFSA (European Food Safety Authority) [8]. The reduction of serum cholesterol could be sustained for 85 weeks, as mid- and long-term studies show [9]. It is important to note that the success of those changes depend on the genetic heritage [6] and its interactions with nutrients [10,11].

*Apolipoprotein A5* gene, in its naturally occurring variants, confers risk for CVD and is associated with high triglyceride levels. Congenital heart diseases and CVD risk are increased when carrying *MTHFR* gene polymorphisms [12], more specifically when carrying the *C677T* polymorphism [13]. Another gene relevant to cholesterol metabolism is the hepatic triglyceride lipase gene (*LIPC*). LIPC not only works as a hydrolase but also as a ligand factor for lipoprotein uptake [14]. *Lipoprotein (a)* is a well-known predictor for stroke, coronary heart disease and other atherosclerotic diseases [15]. This is due to its connection with low-density lipoprotein cholesterol (LDL-cholesterol) [16]. Additionally, *APOE* directly influences the catabolism of lipoproteins [17].

Previous research has been carried out on the effect of variations in genes such as *ABCG5/G8*, *NPC1L1*, *APOA4*, *SR-BI*, *HMG-CoA*, *CETP*, *APOE* and *CYP7A1* in relation to the cholesterol-lowering response to plant sterols/stanols [18,19,20,21,22]. In addition, several reviews have addressed the impact of genes on the cholesterol-lowering efficacy of plant sterols/stanols [23].

The aim of this trial was to determine whether the intake of 2.2 g of PS in milk would influence cholesterol levels in children, according to different genetic polymorphisms.

## 2. Materials and Methods

### 2.1. Study Design

This pilot study was designed as an interventional study. Participants were recruited at Hospital El Escorial in Madrid. Parents’ consent and children’s assent were obtained before the commencement of the trial.

Naturcol milk with PS (Corporación Alimentaria Peñasanta, S.A., Spain) was supplied to the participants throughout the study. Naturcol milk nutritional composition, per 250 mL, was: 36 kcal, 0.5 g fat, 0.2 g saturated fat, 4.7 g carbohydrates of which 4.7 g were sugars, 3.2 g protein, 0.13 g salt, and 120 mg calcium.

**Figure 1:**
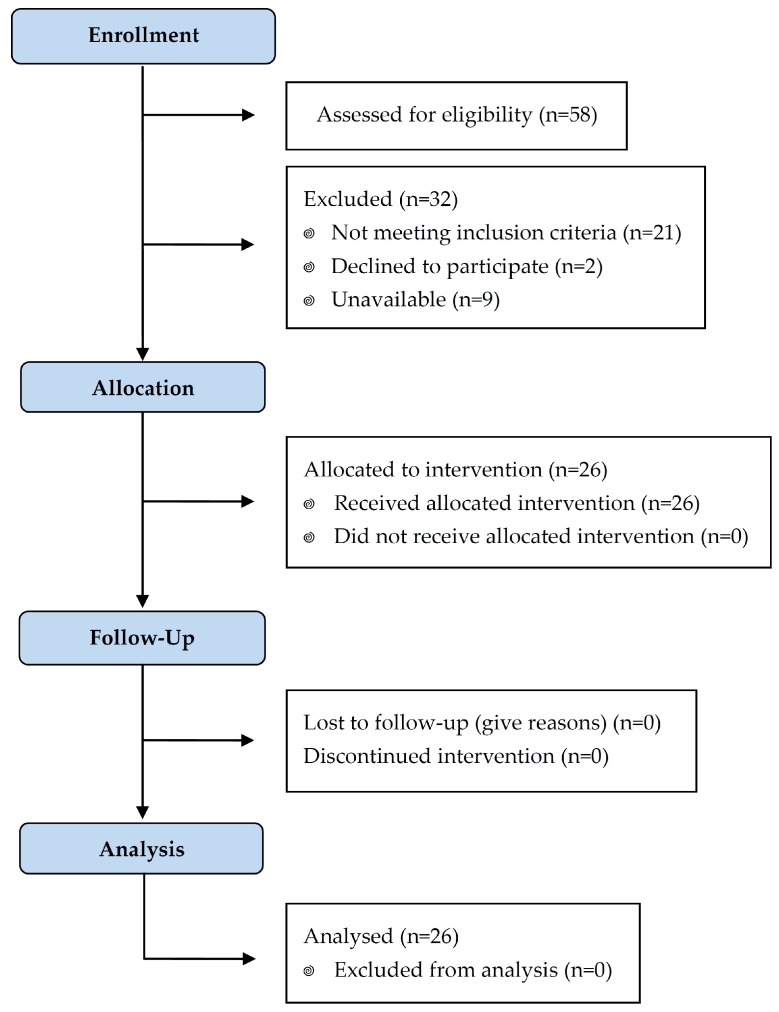
Flow diagram of study design.

Two glasses, of 350 mL capacity each, were ingested by each participant on a daily basis for 3 weeks. The participants had a daily consumption of 2.24 g of PS, contained in 700 mL of regular skim milk. To be eligible for analysis, compliance had to be met and was defined as a minimum consumption of 80% according to empty milk packages returned. Blood tests were performed before and after the intervention (see Figure 1).

A total of 58 participants, 22 boys and 36 girls with a mean age of 8.82 ± 2.28 y were enrolled. Total cholesterol was used as a primary biomarker, based on the clinical practice guidelines by the Spanish Pediatrics Association (AEP) [24], to recruit participants.

### 2.2. Inclusion and Exclusion Criteria

Inclusion criteria: 5 to 12 years-old children, with a total cholesterol (TC) >170 mg/dL and/or LDL-cholesterol >110 mg/dL.

Exclusion criteria: TC <170 mg/dL and/or LDL-cholesterol <110 mg/dL, pubertal development at the beginning of the trial (Tanner Stage II), intolerance to lactose, allergic to proteins originating from cow’s milk, galactosemia, celiac disease, any known chromosomopathies and growth hormone therapy for short stature.

### 2.3. Clinical Analyses

Blood samples were taken at Hospital El Escorial in San Lorenzo de El Escorial (Madrid, Spain) by trained personnel. Participants were asked to fast 12 h prior to the extraction.

Blood was collected in gel serum tubes with an s-Monovette in aspiration [25,26,27]. Blood samples were refrigerated at 5 ± 3 °C after extraction and during shipment to the laboratory. Samples were centrifuged at 1200× *g* for 10 min at 20 ± 5 °C. The stability of the samples was 1 week at 5 ± 3 °C.

Serum total cholesterol (TC) was determined after enzymatic hydrolysis and oxidation, indicator quinoneimine is formed from hydrogen peroxide and 4-aminoantipyrine in the presence of phenol and peroxidase. Analytical ultracentrifugation with density gradient flotation was used to isolate LDL-cholesterol and HDL-cholesterol subfractions, simultaneously. Non–HDL-C was calculated by the following equation: (non-HDL-cholesterol = TC − HDL-cholesterol).

### 2.4. Genetics

The response level to the hypercholesterolemia plant sterol treatment according to different genetic polymorphisms was studied by means of genomic analysis. The different genetic polymorphisms studied were PPARα L162V, LIPC C-514T, APOE APOE2/3/4, APOE 2,3,4 APOA5 C56G Ser19Trp, APOA5 1131T>C, MTHFR C677T, Prothrombin G20210A, F5 Arg506Gln, and LPA I4300M. Out of 58 participants, 26 were recruited for the genetic test: 10 boys and 16 girls with a mean age of 8.7 ± 2.06 years.

Genomic DNA was extracted from saliva samples, for genotyping the single nucleotide polymorphism (SNP) using the Biobank Axiom1 96-Array from Affymetrix. Genotype calling was performed as stated in Affymetrix’s best practice guidelines, including analysis with SNPolisher, assuming a quality control rate of >0.97 [28,29].

To extract saliva-derived DNA samples under high molecular weight and high-quality conditions, each sample was run on an agarose gel. Then, the optical density of the extracted DNA (OD260/280 ratio) was analyzed at 260 nm and 280 nm wavelengths to ensure maximum purity of the extracted DNA (ratio >1.7). Duplicate analyses were performed to obtain precise and reliable results.

### 2.5. Study Variables

Anthropometric measurements, including weight (kg), height (m), Body Mass Index (BMI) (kg/m^2^), total body fat (%), visceral fat (%) and lean body mass (kg), were taken by trained staff who used precise measuring techniques. An electrical bioimpedance InBody Model 270, tetrapolar multi-frequency (20 and 100 kHz), was used to measure weight, BMI and body composition. Manufacturer’s recommendations and a standard protocol were followed to undertake the analysis.

Anthropometric measurements, along with other study variables such as age, sex, health habits, sleep quality, clinical and pharmacological history, use of tobacco and alcohol, food consumption frequency, intestinal transit and physical activity, were recorded in an ad hoc questionnaire designed for this study. The analytical markers of interest obtained from the blood analysis were total cholesterol, high-density lipoprotein cholesterol (HDL-cholesterol), LDL-cholesterol, and non-HDL-cholesterol. Confounding factors were also considered with an affinity table after ingestion (>95%), monitoring of the non-modification of baseline habits during the trial, and a record of food consumption frequencies to control the ingestion of foods that may influence the metabolism of cholesterol upwards or downwards.

### 2.6. Statistical Analysis

Statistical analysis of the data collected was run using the SPSS^®^ (v. 21) statistical software (IBM Corp., Armonk, NY, USA). Descriptive analysis of the sociodemographic variables, anthropometric measurements, and the decrease percentage from baseline to final measures of lipid values after the ingestion of Naturcol were included. The Shapiro–Wilk test was used to determine lipid values normality. Student’s T-test, for paired samples, or the Wilcoxon rank sum test according to compliance with the assumption of normality of the dependent variables was applied to analyze the efficacy of the consumption of Naturcol. The effect size and the proportion of the mean differences were estimated regarding the standard deviation of the baseline, or milk with PS. A value of *p* < 0.05 was considered a significant difference. The statistical power was set at 90%. Distributions calculation of the usual genetics was not taken into account, since genetic studies need larger samples to be carried out.

### 2.7. Ethical Standards

This study followed the ethical principles stated in the Helsinki Declaration. All procedures involving human subjects/patients were approved by the Bioethics Committee of Hospital Universitario Puerta de Hierro Majadahonda, Majadahonda, Madrid, Spain. Written informed consent was obtained from all subjects.

## 3. Results

Twenty-six subjects (16 girls, 10 boys) completed the trial. The remaining 32 subjects did not meet the inclusion criteria (*n* = 21), declined to participate (*n* = 2) or were not available when needed (*n* = 9). They had an average age of 8.7 ± 2.06 years and weighed 33.08 ± 13.00 kg (BMI 18.79 ± 4.20 kg/m^2^) (Table 1). Baseline TC was 236.6 mg/dL, LDL-cholesterol was 157.3 mg/dL and HDL-cholesterol 58.2 mg/dL. Demographic variables did not differ significantly between subjects.

In Table 2, the descriptive statistics of genes and haplotypes can be found.

No statistically significant differences (*p* > 0.05) were seen between genotypes of *APOEA5 MTHFR C677T, PPAR_ALPHA L162V*, and *APOE APOE2, 3, 4* genes and the lower percentage of lipid parameters (TC, HDL-cholesterol, and non-HDL-cholesterol) (Table 3).

No statistically significant differences (*p* > 0.05) were seen in genotypes *LIPC C-514T* and HDL-cholesterol and *PPAR-alpha L162V* and TC and HDL-cholesterol. *LIPC C-514T* showed statistically significant changes for the levels of TC (*p* = 0.0414), LDL-cholesterol (*p* = 0.004) and non-HDL-cholesterol (*p* = 0.010); *PPAR-alpha L162V* showed a significant difference for the levels of LDL-cholesterol and non-HDL-cholesterol *(p* = 0.013; *p* = 0.013), respectively (Table 3).

Since genes *Prothrombin G20210A* and *F5 Arg506Gln* only had one haplotype, an analytical study could not be applied. Even though there are visible differences in HDL-cholesterol and LDL-cholesterol, Table 3 shows a lack of statistical significance for many of the analysed genes. These could be due to the uneven frequency of the haplotypes in this sample.

All of the participants analyzed complied with the treatment under the guidelines given and none of them incurred adverse events.

## 4. Discussion

Plant sterols are known to compete with cholesterol for incorporation into micelles in the intestine and hence in the presence of plant sterols less cholesterol can be transported in micelles leading to a lesser absorption through the gut wall [30] without changing HDL-cholesterol levels. The efficacy of phytosterols to reduce significantly the serum LDL-cholesterol when incorporated into foods and supplements is well-documented [31]. An analysis of PS added to different food matrices was carried out by Clifton et al. (2004) [32]. They reached the conclusion that milk is the matrix with better results in reducing LDL-cholesterol. However, Demonty et al. (2009) [9] showed that there is no difference in the cholesterol-lowering efficacy between different food formats.

The use of PS for the treatment of CVD risk biomarkers has been covered extensively by recent scientific literature [33,34,35,36].

*APOA5T-1131C* is a risk factor for stroke disease sufferers, independent of the subgroup. More precisely, the C allele variant of *APOA5T-1131C* is a risk factor for ischemic stroke and heart disease [37,38], and it increases triglyceride levels in serum. This polymorphism is strongly associated with coronary heart disease risk [39]. However, the 56G allele is only recognized as a risk factor for large-vessel-associated stroke, according to Maasz et al. (2008) [35]. Non-carriers of the minor allele benefit from lower very low-density lipoprotein cholesterol (VLDL-cholesterol) concentrations (0.6 ± 0.22 mmol/L) than the carriers of the minor allele, who present higher concentrations (0.7 ± 0.32 mmol/L) (*p* = 0.01) [39].

Carriers of the ε4 allele of the *ApoE4* have a higher risk of suffering coronary heart disease. On the contrary, *ApoE2* polymorphisms are not significantly related to coronary risk [40].

There seems to be an inverse relationship between LIPC and HDL-cholesterol [41], even though its influence has not been clearly described. Since the metabolism of glycerophospholipids is influenced by the hepatic lipase gene, it is believed that it may also alter HDL-cholesterol plasma concentrations [42]. As suggested by Posadas-Sanchez et al. [43], the TT genotype of *LIPC C-154T* polymorphism is related to higher levels of triglycerides/HDL-cholesterol index (*p* = 0.046) and triglycerides (*p* = 0.0002), under a dominant model. Since the metabolism of glycerophospholipids is influenced by the hepatic lipase gene, it is believed that it may also alter HDL-cholesterol plasma concentrations [42]. As suggested by Posadas-Sanchez et al. [43], the TT genotype of *LIPC C-154T* polymorphism is linked with increased levels of triglycerides/HDL-cholesterol index (*p* = 0.046) and triglycerides (*p* = 0.0002), under a dominant model. Wang et al. [44], on the other hand, associated the same genotype to the presence of LDL-cholesterol (*p* = 0.003). The *LIPCC-514T* polymorphism was involved with hypertriglyceridemia (OR = 1.36, *p* = 0.006) and coronary artery calcification (OR = 1.44, *p* = 0.015), under a dominant model. In addition, obese boys and non-obese girls carrying the T allele presented higher levels of LDL-cholesterol and TC and LDL-cholesterol, respectively (all *p* < 0.05).

Finally, carriers of the CT allele of the *C677T* polymorphism present a higher risk of congenital heart disease compared to the wild CC genotype (OR = 2.249, 95% CI 1.305–3.877, *p* = 0.003) [41]. Besides, the risk of congenital heart disease is significantly associated with the homozygous mutant genotype TT (OR = 3.121, 95% CI 1.612–6.043, *p* = 0.001) [39].

Studies with children show controversial results with *LIPC C-514T*. Riestra et al. (2009) [42] described a relationship between *LIPC* and HDL-cholesterol levels modulated by fat intake in children. Agirbasli et al. (2013) [45] on the contrary, could not find an association between *LIPC* and the lipidic profile in a Turkish cohort.

In Chinese children, a link between the TT haplotype of *LIPC C-514T* was related with an increase in triglycerides, TC, LDL-cholesterol and HDL-cholesterol levels. It should be noticed that this effect depended on the presence of obesity and male gender [44].

SNP within the *CYP7A1* and *ApoE* genes, as well as possibly genes including *ABC G5* and *G8*, have been found to be predictors of the LDL-cholesterol response to plant sterol/stanol treatment. However, this study found that other gene variants than those previously described seem to affect the response of plant sterols on serum lipids. It is worth noting that nutraceuticals can go beyond the genetic influence, they may influence lipid levels beyond the skills in managing sterols [46].

### Limitations

This was a pilot-type study with a rather small number of children participating and a short duration of just eight weeks intervention. Other genotypes reported before to have an impact where not studied.

## 5. Conclusions

The results of this study show that only *LIPC C-514T* and *PPAR-alpha L162V* show a statistically significant effect on the lipidic profile. This could be due to the small size of our sample and the uneven distribution of the genetic haplotypes reviewed in this clinical trial.

Future research with bigger samples is needed to understand the interplay between genomics and cardiovascular health.

The reader should be aware that results cannot be extrapolated to the Spanish population, due to the small sample size. A smaller, humble pilot study was carried out since a bigger sample size and more sophisticated research would incur higher costs.

## Figures and Tables

**Table 1:** Descriptive statistics of the anthropometric measurements and lipid profile.

	Total (*n* = 26)		Males (*n* = 10)		Females (*n* = 16)	
	Mean	SD ^a^	Mean	SD	Mean	SD
Age (years)	8.7	2.06	8.5	2.44	8.8	1.89
Weight (kg)	33.1	13.00	30.1	10.96	35.3	14.39
Height (m)	1.3	0.12	1.3	10.13	1.3	0.12
BMI b (Kg/m^2^)	18.8	4.20	17.2	3.18	20.0	4.59
Body fat (%)	25.5	9.66	20.8	6.99	29.0	10.16
Visceral fat (kg)	3.8	4.04	2.4	2.83	5.0	4.54
Muscle (kg)	11.5	4.46	11.1	4.1	11.9	4.86
	**Change from Baseline (%)**					
Total cholesterol	−12.1	−9.71	−12.5	−7.52	−12.2	−12.62
LDL ^c^-cholesterol	−16.2	−12.78	−17.3	−11.95	−3.2	−16.42
HDL ^d^-cholesterol	−2.1	−14.44	3.3	−13.19	−15.9	−15.75
Non-HDL-cholesterol	−15.9	−11.66	−17.3	−10.90	−15.6	−14.47

^a^ SD, standard deviation; ^b^ BMI, body mass index; ^c^ LDL-cholesterol, low-density lipoprotein cholesterol; ^d^ HDL-cholesterol, high-density lipoprotein cholesterol.

**Table 2:** Descriptive statistics of genes and haplotypes.

Gene	Haplotype	Frequency (*n*)	Percentage (%)
*APOA5 C56G Ser19Trp (rs3135506)*	CG	5	19.2
	GG	21	80.8
*MTHFR C677T (rs1801133)*	CC	8	30.8
	CT	15	57.7
	TT	3	11.5
*LIPC C-514T (rs1800588)*	CC	6	23.1
	CT	13	50
	TT	7	26.9
*LPA I4300M (rs3798220)*	TT	25	96.2
	TC	1	3.8
*PPAR-alpha L162V (rs1800206)*	CC	22	84.6
	CG	4	15.4
*APOA5 1131T > C (rs662799)*	TT	24	92.3
	TC	2	7.7
*APOE Haplotype APOE2/3/4 (rs429358)*	TT	22	84.6
	TC	4	15.4
*APOE Haplotype APOE2,3,4 (rs7412)*	TC	5	19.2
	CC	21	80.8

**Table 3:** Mean changes (in percent) of serum lipids after the plant sterol intervention.

Genes		Change from Baseline (%)			
		HDL-c ^a^	Total	LDL-c ^b^	No-HDL-c ^c^
*APOA5 C56G*	CG	−0.30	−10.07	−15.98	−13.78
*Ser19Trp*	GG	−0.96	−12.8	−16.53	−16.75
	CC	9.76	−10.60	−17.04	−17.71
*MTHFR C677T*	CT	−6.33	−14.60	−18.21	−17.46
	TT	−3.40	−5.25	−6.39	−1.93
	CC	3.10	−5.33 *	−4.17 *	−5.58 *
*LIPC C-514T*	CT	−0.03	−11.45 *	−15.97 *	−15.67 *
	TT	−5.12	−19.82	−26.00	−24.86
*LPA I4300M*	TT	−0.29	−12.90	−17.49	−17.39
	TC	−13.73	2.92	9.18	10.00
*PPAR-alpha L162V*	CC	−1.45	−13.74	−18.44 *	−18.23 *
	CG	3.70	−4.33	−1.62 *	−2.36 *
*APOA5 1131T > C*	TT	−0.38	−11.84	−16.01	−15.75
	TC	−5.99	−17.67	−21.08	−21.77
*APOE Haplotype APOE2/3/4*	TT	0.93	−11.58	−15.72	−15.97
	TC	−13.70	−16.22	−21.58	−18.23
*APOE Haplotype APOE2,3,4*	TC	−12.32	−16.29	−21.18	−18.71
	CC	1.36	−11.34	−15.51	−15.76

^a^ HDL-c, high-density lipoprotein cholesterol; ^b^ LDL-c, low-density lipoprotein cholesterol; ^c^ no-HDL-c, non-high-density lipoprotein cholesterol. * Results with an asterisk indicate a *p*-value < 0.05.
